# Rare case of nephrocalcinosis in a 14-year-old girl: Questions

**DOI:** 10.1007/s00467-016-3434-1

**Published:** 2016-07-06

**Authors:** Omar Bjanid, Piotr Adamczyk, Małgorzata Stojewska, Dagmara Roszkowska-Bjanid, Magdalena Paszyna-Grześkowiak, Agnieszka Jędzura, Joanna Oświęcimska, Katarzyna Ziora, Aurelia Morawiec-Knysak, Maria Szczepańska

**Affiliations:** 1 Department of Pediatric Nephrology with Dialysis Division for Children, Public Clinical Hospital No. 1, ul. 3 Maja 13/15, 41-800 Zabrze, Poland; 2 Chair and Clinical Department of Pediatrics, School of Medicine with the Division of Dentistry in Zabrze, Medical University of Silesia in Katowice, ul. 3 Maja 13/15, 41-800 Zabrze, Poland

**Keywords:** Nephrocalcinosis, Autoimmune polyendocrine syndrome type 1, Hypoparathyroidism, Children

## Abstract

A 14-year-old Caucasian girl with a history of primary hypoparathyroidism and unstable calcium and phosphorus levels and on ongoing treatment was admitted to the Department of Pediatric Nephrology because of the onset of nephrocalcinosis and difficulties achieving normocalcemia. Coexistence of hypoparathyroidism, oral candidiasis, dental enamel hypoplasia, and subclinical Hashimoto’s disease was strongly suggestive for autoimmune polyglandular syndrome (APS) type I. One of the clinical implications of this diagnosis is the high probability of future occurrence of adrenal insufficiency and hence the importance of maintaining a high level of suspicion in case of the onset of symptoms like weakness, fainting, hypotonia, or hyperkaliemia. Addison’s disease would, in fact, be quite challenging for the future management of this patient.This clinical quiz highlighted the importance of careful evaluation of all multiorgan symptoms occurring in a patient to prevent further complications.

## Case presentation

**Fig. 1:**
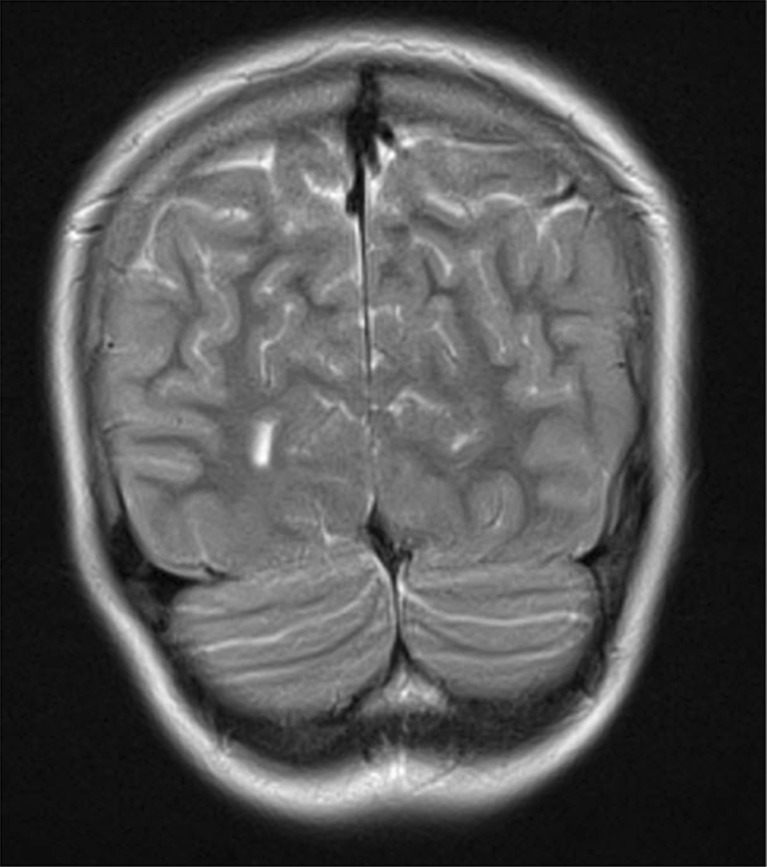
Calcifications in basal ganglia on computed tomography

A 14-year-old Caucasian girl with a history of primary hypoparathyroidism and unstable calcium and phosphorus levels and on ongoing treatment was admitted to the Department of Pediatric Nephrology because of the onset of nephrocalcinosis and difficulties achieving normocalcemia. The obstetric, neonatal, and developmental history was unremarkable. The child was born at full term after a normal seventh pregnancy and fifth delivery, with a birth weight of 3150 g and an Apgar score of 10. An adenoidectomy was performed at the age of 6 years. The first symptoms of the disease appeared at the age of 9 years and consisted of several episodes of syncope and seizures. At presentation, apart from a white-coated tongue, dental caries, and enamel hypoplasia, the clinical, neurological, cardiovascular, and ophthalmic examinations were normal. Laboratory tests revealed hypocalcemia, hyperphosphatemia, and low serum parathyroid hormone (sPTH). Computed tomography (CT) and magnetic resonance (MR) imaging revealed bilateral calcifications in the basal ganglia and the frontal lobes (Fig. 1). The thyroid gland was slightly heterogeneous on ultrasound, and antithyroid peroxidase (anti-TPO) antibodies were present, without any other clinical or biochemical features of hypothyreosis. A treatment of primary hypoparathyroidism based on calcium supplementation and 1-α-hydroxycholecalciferol administration was prescribed. During a period of 3 years (2011–2014), the dosage had to be progressively increased due to persisting hypocalcemia, to a maximum of 4.0 g of calcium carbonate and 1.75 μg of 1-α-hydroxycholecalciferol daily. In January 2015, the patient was hospitalized because of dyspnea, generalized weakness, polydipsia, nausea, and tachycardia. She was severely hypercalcemic at that moment but recovered rapidly after intensive fluid therapy, loop diuretics, and temporary withdrawal of the vitamin D and calcium. The doses were ultimately reduced to 1.25 μg and 3.0 g, respectively. Renal sonography remained normal. In September 2015, however, features of mild nephrocalcinosis appeared, with increased echogenicity of the borders of the renal pyramids, which led to her referral to the nephrology department. On admission, the girl was in good general condition. Physical examination showed the same oral findings as described above, with the confirmation of candidiasis in swab culture. Laboratory tests showed biochemical findings similar to the aforementioned: low level of s PTH, 25-OH-vitamin D, and alkaline phosphatase; hyperphosphatemia; and clearly decreased levels of total and ionized calcium. Persistent hypercalciuria was observed, with an elevated calcium-to-creatinine ratio in a morning urine sample of 0.24 mg/mg, and a decreased magnesium-to-calcium ratio of 0.25 mg/mg. The other results were within the normal range, including an estimated glomerular filtration rate (eGFR) by Schwartz formula of 92 ml/min/1.73 m^2^. A bone densitometry (total body and lumbar region) was appropriate for age. During hospitalization, the girl reported transient acute pain in her right knee. X-ray imaging showed no pathological changes. Ultrasound examination revealed, besides mild nonprogressive bilateral nephrocalcinosis, a duplex left kidney with a mild dilatation of the upper-pole collecting system of 10 mm in sagittal dimension. The findings were scheduled for further observation and treatment.

## Questions

1. What is the final diagnosis?

2. What is the cause of nephrocalcinosis?

3. What further treatment options for the disease are available?

